# Supplementary figures and images for: Human exome and mouse embryonic expression data implicate ZFHX3, TRPS1, and CHD7 in human esophageal atresia
Source: PLoS One. 2020 Jun 5;15(6):e0234246. doi: 10.1371/journal.pone.0234246 (PMC7274392; doi:10.1371/journal.pone.0234246)

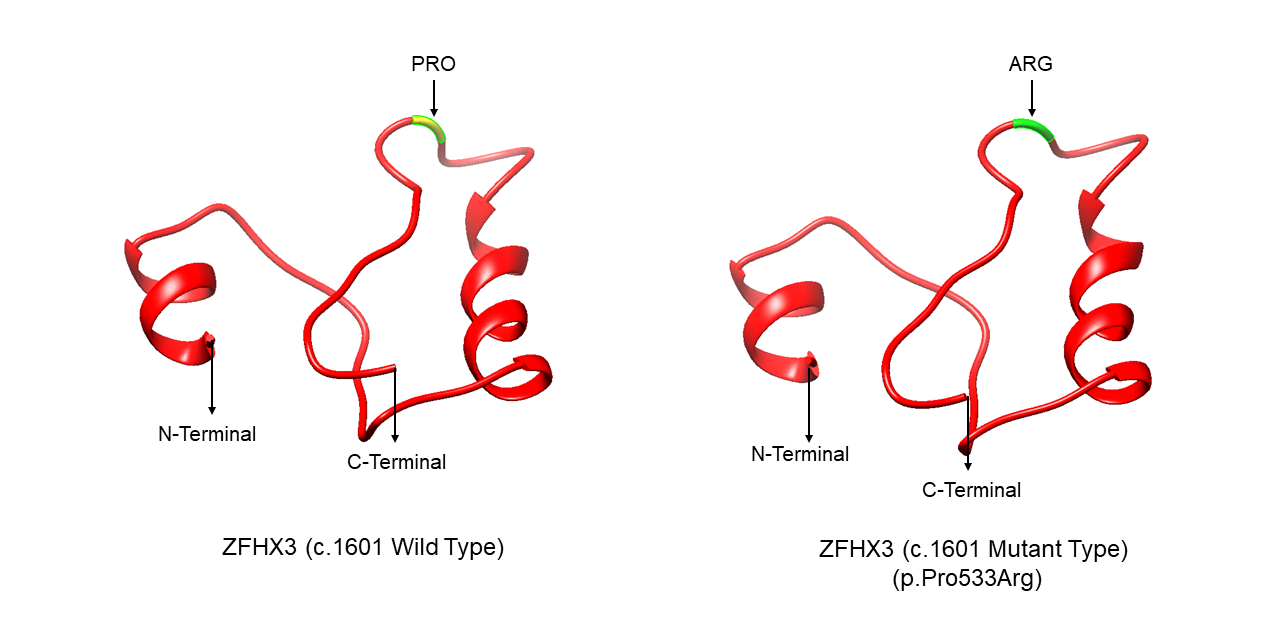

Supplement: S1 Fig — (PNG) [file pone.0234246.s001.png]

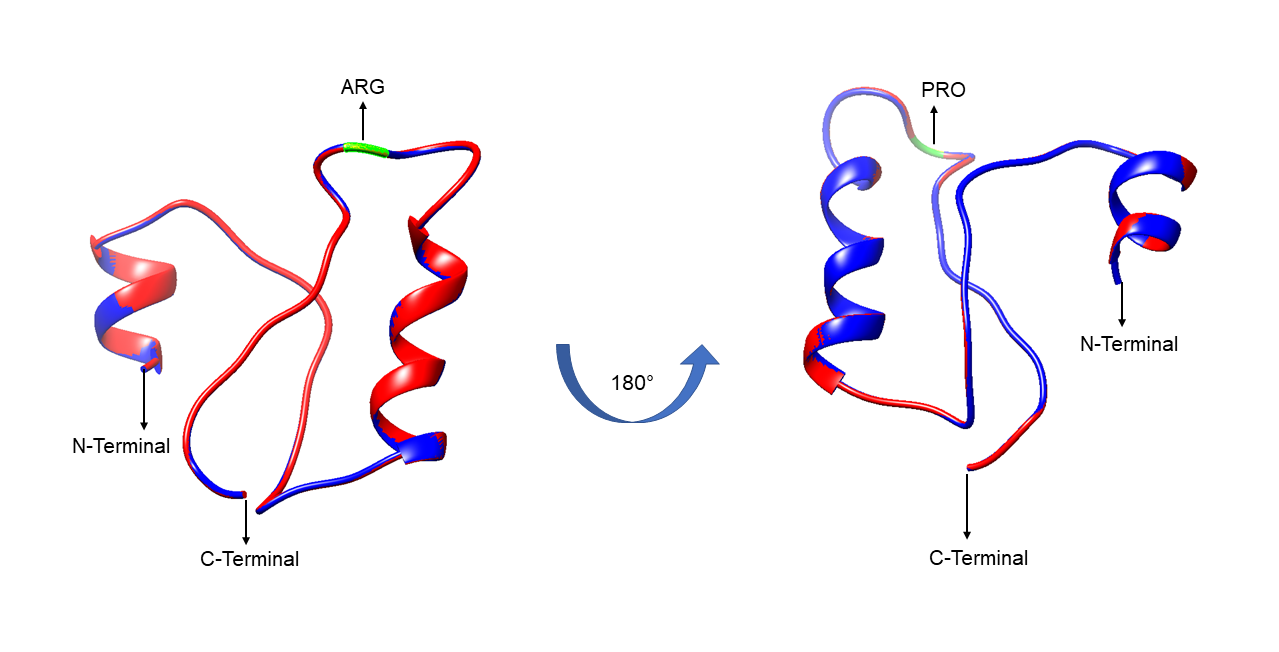

Supplement: S2 Fig — (PNG) [file pone.0234246.s002.png]

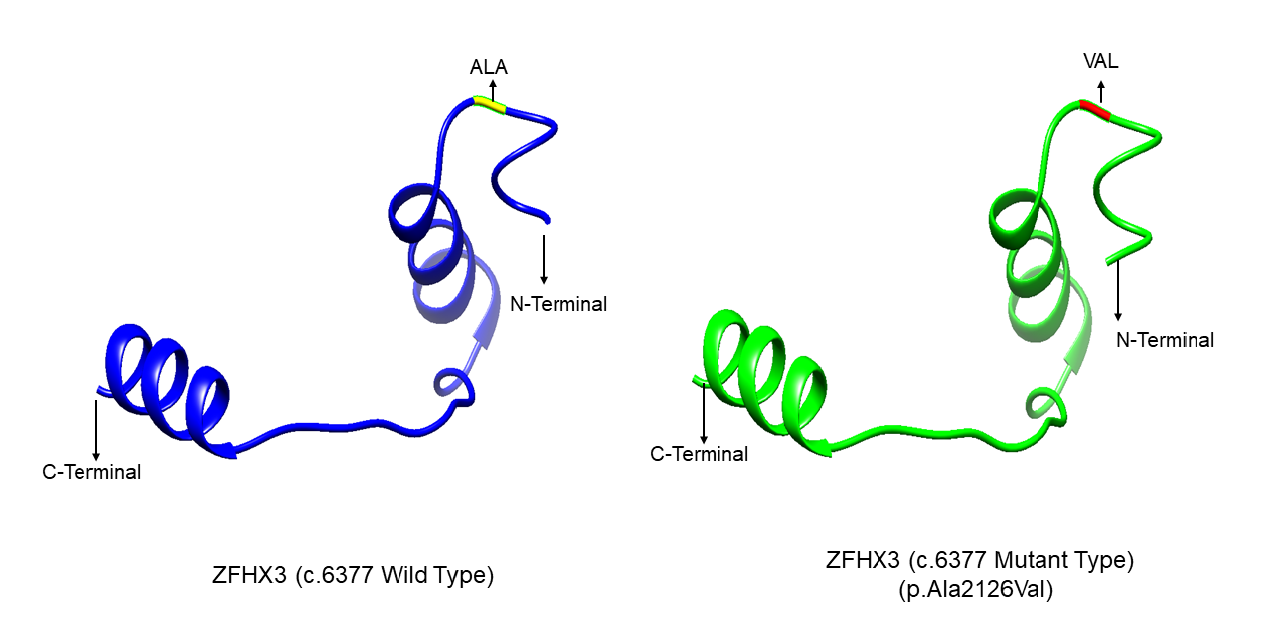

Supplement: S3 Fig — (PNG) [file pone.0234246.s003.png]

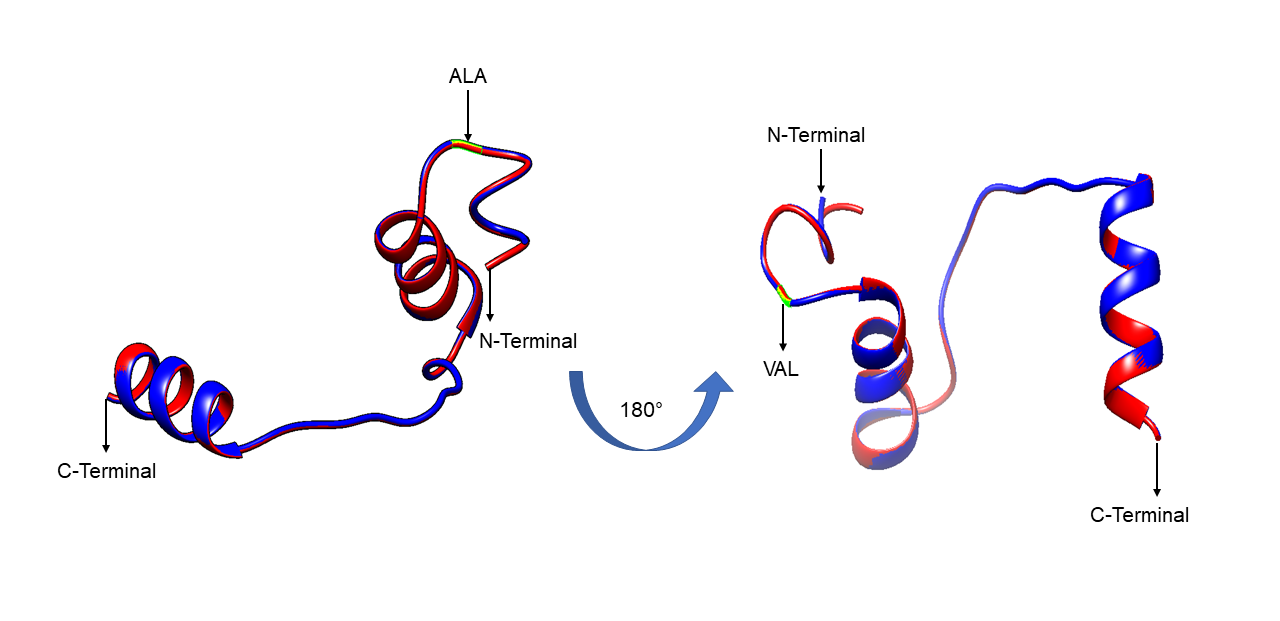

Supplement: S4 Fig — (PNG) [file pone.0234246.s004.png]
