# Supplementary material for: Human exome and mouse embryonic expression data implicate ZFHX3, TRPS1, and CHD7 in human esophageal atresia
Source: PLoS One. 2020 Jun 5;15(6):e0234246. doi: 10.1371/journal.pone.0234246 (PMC7274392; doi:10.1371/journal.pone.0234246)
Supplement: S1 Table — VATER/VACTERL-like association (vertebral defects (V), anorectal malformations (A), cardiac defects (C), tracheoesophageal fistula with or without esophageal atresia (TE), renal malformations (R), and limb defects (L) [5], Ventricular septal defect (VSD), atrial septal defect (ASD). (DOCX) [file pone.0234246.s005.docx]

**Supplemental Table 1 EA/TEF Patients included in WES analysis**

| **Ext.-code** | **Vogt type**  **EA/TEF** | **nonsyndrom /**  **syndromic** | **additional congenital anomalies** |
| --- | --- | --- | --- |
| **2_501** | **IIIb** | **nonsyndromic** | **-** |
| **4_501** | **IIIb** | **syndromic** | **VATER/VACTERL-like association (thoracic vertebral anomalies)** |
| **21_501** | **IIIb** | **nonsyndromic** | **-** |
| **27_501** | **IIIb** | **nonsyndromic** | **-** |
| **34_501** | **IIIb** | **syndromic** | **laryngeal cleft I°, posteriorly rotated ears** |
| **35_501** | **IIIb** | **syndromic** | **VATER/VACTERL-like association (7th cervical vertebral anomaly, dilatation of the ascending aorta, tethered cord)** |
| **36_501** | **IIIb** | **nonsyndromic** | **-** |
| **41_501** | **IIIb** | **syndromic** | **VATER/VACTERL-like association (coarctation of the aorta, unilateral renal agenesis, bilateral undescended testicles)** |
| **46_501** | **IIIb** | **nonsyndromic** | **-** |
| **53_501** | **IIIb** | **syndromic** | **VATER/VACTERL association (choanal atresia, laryngial cleft I°, VSD, cloacal anomaly, caudal regression syndrome, uterus duplex)** |
| **63_501** | **IIIb** | **syndromic** | **VATER/VACTERL-like association (VSD, aberrant subclavian artery, spina bifida occulta, sacral dysplasia)** |
| **75_501** | **IIIb** | **syndromic** | **VATER/VACTERL-like association (ASD, VSD)** |
| **88_501** | **IIIb** | **nonsyndromic** | **-** |
| **90_501** | **IIIb** | **syndromic** | **VATER/VACTERL-like association (malposition of the heart, unilateral cleft lip)** |
| **103_501** | **IIIb** | **nonsyndromic** | **-** |
| **112_501** | **IIIb** | **nonsyndromic** | **-** |
| **113_501** | **IIIb** | **nonsyndromic** | **-** |
| **134_501** | **IIIb** | **nonsyndromic** | **-** |
| **137_501** | **IIIb** | **syndromic** | **VATER/VACTERL association (radial aplasia, thumb hypoplasia, hydronephrosis left sided, anular pancreas)** |
| **141_501** | **IIIc** | **syndromic** | **VATER/VACTERL-like association (unilateral hydronephrosis)** |
| **154_501** | **IIIb** | **syndromic** | **VATER/VACTERL association (perineal fistula, ASD, anular pancreas, coloboma, malformation of the ribs, premature pubic hair)** |
| **167_501** | **IIIb** | **nonsyndromic** | **-** |
| **170_501** | **IIIb** | **syndromic** | **VATER/VACTERL association (ASD, renal malformation, thumb hypoplasia)** |
| **171_501** | **IIIb** | **syndromic** | **palpebral fissure, Meckel diverticulum, umbilical hernia, hip dysplasia** |
| **172_501** | **IIIb** | **syndromic** | **VATER/VACTERL-like association (ASD, cafè-au-lait spots)** |
| **174_501** | **IIIb** | **nonsyndromic** |  |
| **181_501** | **IIIb** | **nonsyndromic** |  |
| **199_501** | **IIIb** | **syndromic** | **VATER/VACTERL association (vertebral defects, VSD, abdominal situs inversus, patent left upper vena cava, kinking of the aorta)** |
| **211_501** | **IIIb** | **nonsyndromic** |  |
| **288_501** | **IIIb** | **syndromic** | **VATER/VACTERL association (VSD, ASD, hydronephrosis, club feet, muscular hypotonia)** |
| **750_501*** | **IIIb** | **syndromic** | **VATER/VACTERL association (anorectal malformation, ASD, renal agenesis right sided)** |
