# Supplementary material for: Human exome and mouse embryonic expression data implicate ZFHX3, TRPS1, and CHD7 in human esophageal atresia
Source: PLoS One. 2020 Jun 5;15(6):e0234246. doi: 10.1371/journal.pone.0234246 (PMC7274392; doi:10.1371/journal.pone.0234246)
Supplement: S2 Table — (DOCX) [file pone.0234246.s006.docx]

| **Ext-Code** | **Variant** | **HGNC** | **MutCDNA** | **gnomAD (MAF)** | **SIFT** | **LRT** | **Mutation**  **Taster** | **Mutation**  **Assessor** | **FATHMM** | **PROVEAN** | **Meta**  **SVM** | **Meta**  **LR** | **Fathmm**  **MKL_coding** | **CADD**  **Score** |
| --- | --- | --- | --- | --- | --- | --- | --- | --- | --- | --- | --- | --- | --- | --- |
| 4_501 | 1 | *EEF1D* | c.874C>T |  | - | N | A | - | - | - | - | - | N | **28,5** |
|  | 2 | *CELSR1* | c.4357G>A | 3/282,594 (0.00001) | T | U | N | N | T | N | T | T | D | 15,3 |
| 21_501 | 3 | *HPS3* | c.1189C>T | 10/282,776 (0.00004) | D | D | A | M | T | D | D | T | D | **35** |
| 27_501 | 4 | *PIGC* | c.716C>T |  | T | D | D | M | T | N | T | T | D | 11,6 |
| 35_501 | 5 | *NFX1* | c.1723G>A |  | D | N | N | L | T | N | T | T | D | **20,8** |
| 36_501 | 6 | *ZFHX3* | c.1601C>G |  | D | N | D | L | T | N | T | T | D | **22,3** |
| 41_501 | 7 | *MTA3* | c.393C>A | 1/237,600 (0.000004) | **D** | **D** | **D** | H | **D** | **D** | **D** | **D** | **D** | **26** |
| 46_501 | 8 | *FANCB* | c.782G>A |  | T | N | N | N | T | N | T | T | N | 7,2 |
|  | 9 | *PLEC* | c.6704G>A | 17/272,690 (0.00006) | D | U | D | N | T | N | T | T | D | **26,5** |
| 63_501 | 10 | *PPIP5K2* | c.686G>A | 2/247,732 ( 0.000008) | D | D | D | M | T | D | T | T | D | **34** |
| 88_501 | 11 | *CLP1* | c.814C>A | 1/251,486 (0.000003) | T | D | D | L | T | N | T | T | D | 17,4 |
|  | 12 | *GPR133* | c.1033G>A | 6/282,534 (0.00002) | T | N | N | N | T | N | T | T | N | 0,016 |
|  | 13 | *SLC5A2* | c.644T>C |  | **D** | **D** | **D** | H | **D** | **D** | **D** | **D** | **D** | **27,6** |
| 90_501 | 14 | *KIAA0556* | c.3730C>T |  | D | N | N | L | T | N | T | T | N | 1,9 |
| 141_501 | 15 | *STAB1* | c.6145C>T | 9/278,948 (0.00003) | T | N | N | M | T | D | T | T | N | **24,1** |
| 154_501 | 16 | *GGT6* | c.1045A>G |  | D | N | N | N | T | D | T | T | N | 5,9 |
| 167_501 | 17 | *CHD7* | c.4187C>G |  | **D** | **D** | **D** | H | T | **D** | **D** | **D** | **D** | **33** |
| 172_501 | 18 | *NPR2* | c.952C>G |  | T | N | D | L | D | D | T | T | D | **22,2** |
| 174_501 | 19 | *UBA3* | c.1088C>T |  | D | D | D | L | T | D | T | T | D | **27,8** |
| 181_501 | 20 | *TANC2* | c.2357C>T |  | T | D | D | L | T | D | T | T | D | **22,7** |
| 288_501 | 21 | *TRPS1* | c.1630C>T |  | - | D | A | - | - | - | - | - | D | **36** |
|  | 22 | *APOL2* | c.319G>C |  | T | N | N | N | T | N | T | T | N | 0,004 |
| 750_501* | 23 | *ZFHX3* | c.6377C>T | 5/250,880 ( 0.00002) | **D** | **D** | **D** | L | T | **D** | **D** | **D** | **D** | 19,2 |
