# Supplementary material for: Human exome and mouse embryonic expression data implicate ZFHX3, TRPS1, and CHD7 in human esophageal atresia
Source: PLoS One. 2020 Jun 5;15(6):e0234246. doi: 10.1371/journal.pone.0234246 (PMC7274392; doi:10.1371/journal.pone.0234246)
Supplement: S1 Data — (DOCX) [file pone.0234246.s007.docx]

**Material and methods**

***In silico* prediction tools**

By using SIFT, variants are predicted as “D (damaging)”, otherwise as “T (tolerated)” based on position-specific scoring matrix. In LRT program, which is based on likelihood ratio test of codon neutrality: “D” stands for deleterious, “N” represents neutral and “U” stands for unknown. The variants are reported as “A (disease causing automatic)”, “D (disease causing)”, “N (polymorphism)” or “P (polymorphism automatic)” according to the prediction tool MutationTaster. Mutation Assessor takes into account combinatorial entropy formalism and predicts the functional impact of the variants: predicted functional “H (high)” or “M (medium)” and non-functional “L (low)” or “N (neutral)”. Based on FATHMM, which is on the basis of Hidden Markov models, a variant is predicted as “D (damaging)” or “T (tolerated)”. PROVEAN is based on delta alignment score and provides binary predictions: “D (damaging)” and “N (neutral)”. MetaSVM and MetaLR are based on “radial kernel support vector machine” and “logistic regression” representatively, and both of them predict a variant as “D (damaging)”, otherwise as “T (tolerated)”. The prediction tool fathmm-MKL coding is based on multiple kernel learning model and also provides binary prediction: “D (damaging)” and “N (neutral)”. The CADD score, based on linear kernel support vector machine, is a tool for predicting the deleteriousness of SNVs (single nucleotide variants) as well as insertion/deletion variants at the genome position as a conservation score. Here we prioritized those variants that are classified to be deleterious by at least eight out of ten in silico prediction tools except for truncating variants as these variants are automatically called deleterious.

**Results**

***De novo* variant in *FANCB***

In patient 46_501 we identified a *de novo* variant in the X-linked *FANCB* gene which has been previously associated with the VACTERL-H phenotype comprising EA/TEF as frequent phenotypic feature (McCauley et al., 2011). However, the here identified variant in *FANCB* has been classified as benign by all nine *in silico* prediction programs used by dbNSFP v3.0. Furthermore, the variant was identified in a female patient (46_501, Table 1) with normal X-inactivation pattern (data not shown). Hence, we classified the variant as not disease causing and thus not responsible for the EA/TEF phenotype of our patient (46_501).

***De novo* variant in *KIAA0556***

In patient 90_501 we identified a novel *de novo* amino acid change p.His1244Tyr in a highly conserved region of *KIAA0556*, a gene previously associated with autosomal recessive inherited Joubert syndrome (OMIM #616784) (Roosing et al., 2016; Sanders et al., 2015). While the expression of Kiaa0556 at days E8.5, E12.5 and postnatal in mouse esophageal tissue is relatively high, the applied *in silico* prediction tools do not support the identified amino acid change p.His1244Tyr as disease causing. Furthermore, the only monoallelic putative damaging variant reported in the literature in *KIAA0556* was described by Hong et al. (Hong et al., 2013) reporting a novel non-synonymous somatic *de novo* change (chr16:27,788,348 G>T) in *KIAA0556* in an imatinib-resistant paravertebral dermatofibrosarcoma protuberans. Hence, we propose that the here identified *de novo* change is not involved in the development of EA/TEF in patient 90_501.

**References**

Hong, J.Y., Liu, X., Mao, M., Li, M., Choi, D.I., Kang, S.W., Lee, J., and La Choi, Y. (2013). Genetic aberrations in imatinib-resistant dermatofibrosarcoma protuberans revealed by whole genome sequencing. PloS One *8*, e69752.

McCauley, J., Masand, N., McGowan, R., Rajagopalan, S., Hunter, A., Michaud, J.L., Gibson, K., Robertson, J., Vaz, F., Abbs, S., et al. (2011). X-linked VACTERL with hydrocephalus syndrome: further delineation of the phenotype caused by FANCB mutations. Am. J. Med. Genet. A. *155A*, 2370–2380.

Roosing, S., Rosti, R.O., Rosti, B., de Vrieze, E., Silhavy, J.L., van Wijk, E., Wakeling, E., and Gleeson, J.G. (2016). Identification of a homozygous nonsense mutation in KIAA0556 in a consanguineous family displaying Joubert syndrome. Hum. Genet. *135*, 919–921.

Sanders, A.A.W.M., de Vrieze, E., Alazami, A.M., Alzahrani, F., Malarkey, E.B., Sorusch, N., Tebbe, L., Kuhns, S., van Dam, T.J.P., Alhashem, A., et al. (2015). KIAA0556 is a novel ciliary basal body component mutated in Joubert syndrome. Genome Biol. *16*, 293.
