# Supplementary material for: Human exome and mouse embryonic expression data implicate ZFHX3, TRPS1, and CHD7 in human esophageal atresia
Source: PLoS One. 2020 Jun 5;15(6):e0234246. doi: 10.1371/journal.pone.0234246 (PMC7274392; doi:10.1371/journal.pone.0234246)
Supplement: Attachment — Submitted filename: R1_Rebuttal_Letter.docx [file pone.0234246.s009.docx]

PONE-D-20-03485R1

We thank the reviewer for his comments. We have addressed this comment below. In the revised manuscript, all changes to the earlier revised version are written in red. We hope that our manuscript will now be considered suitable for publication.

**Reviewer #3:**

**Comment:**

Again, the paper would have been stronger with a mechanistic demonstration of the roles of the genes reported as involved in the development of the foregut.

***Answer*** ***to Comment 1:***

We agree with the reviewer that any molecular genetic finding detected in human individuals with congenital malformations should in the following workup of these findings warrant *in vivo* functional studies, e.g. animal models to provide further evidence for the involvement of the identified molecular genetic findings in the expression of the disease. However, the creation of animal models was beyond the scope of the present study. In order to provide some level of functional evidence, we generated mouse transcriptome data from wildtype mice, in order to see, if the identified potential disease genes are actually expressed during the embryonic critical time frame. Here we were able to show, that Zfhx3 is continuously high expressed during all time points, and that CHD7 and Trps1 are differentially expressed over all three time points.

To address the concern of the reviewer, we have added the following sentence to the end of the discussion: “Overall, interpretation of the data is limited by the lack of animal models, at least for the findings in *CHD7*, *TRPS1,* and *ZFHX3*. To the best of our knowledge, there has no animal model been described that would have investigated embryonic foregut development, when these genes have been deleted. In order to definitely conclude that our findings respectively *de novo* variants in *CHD7*, *TRPS1*, and *ZFHX3* have been directly causative for the EA/TEF phenotype in the respective patients, *in vivo* experiments including animal models would be necessary, which were beyond the scope of our present study.”
